# Piperine protects ovarian follicles and stromal cells against doxorubicin-induced adverse effects in mouse ovaries

**DOI:** 10.5935/1518-0557.20250187

**Published:** 2026

**Authors:** Moisés Bruno Marinho Rocha, Ernando Igo Teixeira de Assis, Venância Antônia Nunes Azevedo, Miguel Fernandes de Lima Neto, Anderson Weiny Barbalho Silva, Alana Nogueira Godinho, Jordânia Marques de Oliveira Freire, Emanoel da Silva Félix, Regislane Pinto Ribeiro, Geovany Amorim Gomes, José Roberto Viana Silva

**Affiliations:** 1 Laboratory of Biotechnology and Physiology of Reproduction (LABIREP), Federal University of Ceará, Sobral, CE, Brazil; 2 Nucleus of Research in Animal Experimentation (NUPEX), Federal University of Ceará, Sobral, CE, Brazil; 3 Center of Exact Science and Technology, State University of Acarau Valley, Sobral, CE, Brazil

**Keywords:** chemotherapy, fertility, gonadal toxicity, ovary, piperine

## Abstract

**Objective:**

This study investigates the effects of Piperine (PIP) on doxorubicin (DOX)-induced changes in mouse ovarian follicles, stromal cells, collagen fibers, and mRNA expression of nuclear factor erythroid 2-related factor (NRF2), superoxide dismutase (SOD), and catalase (CAT).

**Methods:**

The mice were randomly divided into seven groups. In the first three groups, they received saline (1), both DOX and N-acetylcysteine (2), or DOX only (3). In groups 4 and 5, mice were treated with DOX in combination with 0.1 or 10.0mg/kg PIP. In groups 6 and 7, mice received 0.1 or 10.0mg/kg PIP alone. After 10 days, ovaries were collected and used to evaluate follicular morphology and growth, collagen fibers, stromal cells, and mRNA for NRF2, SOD, and CAT.

**Results:**

Mice treated with DOX showed reduced percentage of normal follicles, but the combination of DOX with PIP or NAC prevented this effect, maintaining follicle integrity similar to untreated animals. Ovaries of mice treated with PIP alone had similar percentage of normal follicles compared to control group. Additionally, the association of DOX and PIP preserved collagen levels similar to control, while PIP or NAC alone did not influence collagen distribution. Ovaries of mice treated with both DOX and NAC showed a reduction in stromal cells, but those treated with both DOX and PIP maintained the levels of collagens similar to control.

**Conclusions:**

The DOX and PIP preserved the integrity of follicles and collagen fibers in mouse ovaries, which opens a new possibility to protect primordial follicles during chemotherapy.

## INTRODUCTION

Cancer treatment involves the use of highly potent cytotoxic drugs that damage germ cells, particularly oocytes (Vilar *et al*., 2018). Doxorubicin (DOX) is a drug used to treat a variety of cancers and has multiple mechanisms of action, including DNA intercalation, topoisomerase II inhibition, and oxidative stress (Kciuk *et al*., 2023). Spears *et al*. (2019) reported that DOX causes premature ovarian failure by inducing apoptosis of granulosa cells and death of growing follicles. Recent studies showed 10.0mg/kg DOX increased expression of tumor necrosis factor*-α* and reduced the rate of normal follicles in mice ovaries (Lima Neto *et al*., 2024; de Assis *et al*., 2025). Furthermore, it reduces ovulation rate and ovary size in mice (Ben-Aharon *et al*., 2010) and causes early menopause and increases infertility rate after chemotherapy (Poorvu *et al.*, 2019). In this sense, the protection of ovarian follicular reserve has become one of the main issues to preserve fertility and increase the quality of life of patients (Gao *et al*., 2023). In this context, preserving fertility has become a critical concern for reproductive-age women undergoing chemotherapy. The clinical implications of gonadotoxic treatments extend beyond temporary ovarian suppression, often resulting in premature ovarian insufficiency and permanent infertility (Cacciottola *et al*., 2022). Fertility preservation techniques, such as oocyte and embryo cryopreservation, have emerged as standard strategies prior to chemotherapy initiation. Additionally, ovarian tissue cryopreservation offers an option for prepubertal girls and women who cannot delay treatment (Markowska *et al*., 2024). However, these interventions require specialized infrastructure, are costly, and may not be universally accessible (Melan *et al*., 2018). Therefore, the identification of pharmacological agents capable of reducing ovarian toxicity during chemotherapy, such as natural compounds with antioxidant properties, represents a promising complementary approach to enhance reproductive outcomes and quality of life (Cetinkaya *et al*., 2023).

Piperine (PIP) is a bioactive component existing abundantly in *Piper longum L*. (long pepper) and white or black pepper (*Piper nigrum*) (Li *et al.,* 2019) which has many therapeutic benefits, with analgesic, anti-inflammatory, antioxidant, immunosuppressive, antimicrobial, antihypertensive, antidiabetic and antidepressant effects (Al-Khayri *et al*., 2022; Cinar & Sanlier, 2025). The main effects of this compound are related to protection against oxidative damage by inhibiting or quenching free radicals and reactive oxygen species (ROS) (Rather & Bhagat, 2018). Furthermore, PIP has been shown to reduce oxidative stress and lipid peroxidation in vivo (Saha *et al*., 2022) and to increase superoxide dismutase (SOD) and catalase (CAT) activities in rat gastric tissue (Duan *et al*., 2022). The PIP regulated the expression of nuclear factor erythroid 2-related factor 2 (Nrf2) and Kelch-like ECH-associated protein 1 (Keap1), reducing hyperglycaemia in type 2 diabetic rats (Mounithaa *et al.,* 2023). The PIP also exhibited anti-inflammatory and antioxidant effects in cells treated with lipopolysaccharide (LPS) (Wang-Sheng *et al.,* 2017). The NRF2 signaling pathway is critical for protecting cells from intracellular oxidative stress and inflammation (Mounithaa *et al.,* 2023). Additionally, Fani *et al.* (2024) demonstrated that 10mg/kg PIP mitigated cyclophosphamide-induced testicular histopathological abnormalities, oxidative stress, and apoptosis in mice. The PIP (10mg/kg) also protects rat hippocampal neurons against Kainic acid-induced cytotoxicity by upregulating nerve growth factor expression (Hsieh *et al*., 2022). Thus, it is hypothesized that PIP attenuates the adverse effects of DOX and helps to preserve the integrity of mouse ovarian follicles and stromal cells. This combined treatment may help to preserve the large pool of primordial follicles and help to preserve fertility in patients undergoing chemotherapy.

The aim of this study was to investigate the ability of different doses of PIP (0.1 and 10.0mg/kg) to protect mouse ovaries from DOX-induced damage. The effects of PIP on follicle morphology and development, maintenance of ovarian stromal cell density, collagen fibers, and mRNA levels for NFR2, SOD, and CAT were also evaluated.

## MATERIAL AND METHODS

### Ethics statement

Swiss mice (*Mus musculus*) (n=42) were housed in polyethylene boxes lined with wood shavings (6 animals/box), with free access to filtered water and food, at a temperature of 22±2°C with a 12-hour light/dark cycle. The animals were used according to the guidelines and normative resolutions of the National Council for Control in Animal Experimentation (Brazil). This study was approved by the Institutional Animal Use Ethics Committee (protocol nº 08/21).

### Nomenclature

The tested drugs were doxorubicin (Libbs, Fauldoxo®, doxorubicin hydrochloride, injectable solution, 2 mg/mL) and piperine (TCI - CAS number: 94-62-2, 97%). Piperine was diluted in saline solution supplemented with 0.2% dimethylsulfoxide (DMSO) obtained from Sigma-Aldrich (USA).

### Animals and estrous cycle assessment

Female mice, 2 months old or 18 g in weight, were scored once daily for the estrous cycle over a period of 20 days (Marcondes *et al*., 2002). The stage of the cycle, i.e. proestrus, estrus, metestrus or diestrus, was determined according to the cells observed. Only females with a regular cycle of 4 to 5 days were used in the experiment. Animals with irregular estrous cycle were excluded from the experiment.

### Experimental design

**Figure 1:**
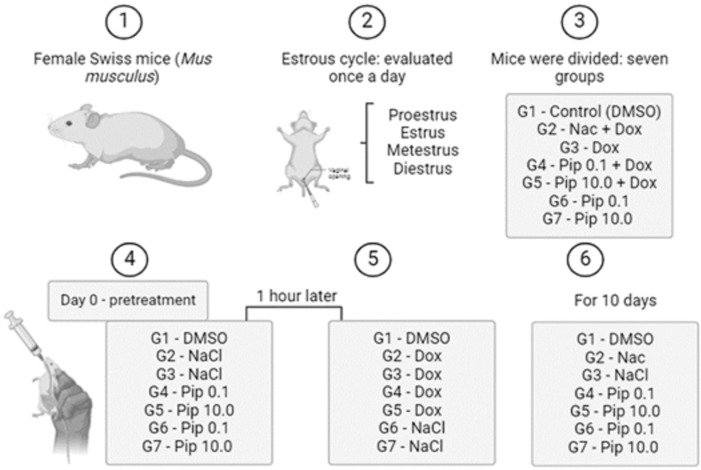
Experimental design.

DMSO: Dimethylsulfoxide; NaCl: Sodium chloride; NAC: N-acetylcysteine; DOX: Doxorrubicin; PIP: Piperine.

Female mice (n=42) were randomly divided into seven groups. In the first three groups, mice received saline solution with 0.2% DMSO (1), both 10.0mg/kg DOX and 150.0mg/kg N-acetylcysteine (2), or 10.0mg/kg DOX only (3). In groups 4 and 5, mice were treated with 10.0mg/kg DOX in combination with 0.1 or 10.0mg/kg PIP. In groups 6 and 7, mice received only 0.1 or 10.0mg/kg PIP (Figure 1). The concentrations of PIP (Fani *et al.,* 2024; Hsieh *et al*., 2022) and DOX (de Assis *et al.,* 2025) were chosen according to previous results of these authors. The DOX administration was performed at the beginning of the experiment and then the mice were treated with saline solution, N-acetylcysteine or PIP by gavage daily for 10 days. The animals were then euthanized and tissues were collected for evaluation of ovarian morphology and gene expression.

### Analysis of ovarian follicle morphology and stromal cell density

Ovaries were fixed in 4% paraformaldehyde for 24 hours, dehydrated in graded series of ethanol, cleared in xylene, and embedded in paraffin. Serial 5-µm thick sections were stained with hematoxylin and eosin and examined under a light microscope (Nikon, Eclipse, TS 100, Japan). The follicles were classified according to their stage of development as primordial or developing follicles, i.e., primary, secondary, and antral follicles. In addition, these follicles were individually classified as morphologically normal if an intact oocyte was present and surrounded by granulosa cells well organized in one or more layers and without pyknotic nuclei. Degenerate follicles were defined as those with a retracted or vacuolated oocyte containing a pyknotic nucleus surrounded by disorganized granulosa cells. To avoid double counting, only follicles in which the oocyte nucleus was visible were counted (Pedersen & Peters, 1968). A total of 150 follicles were evaluated for each group of animals. To assess ovarian stromal cell density, the number of stromal cells in a 100 µm^2^ area was counted. For each treatment, ten fields from different sections of histologic preparations from five different animals were evaluated. The average number of stromal cells per field was calculated as previously described (Cavalcante *et al*., 2019). All evaluations and measurements were performed by a single operator.

### Analysis of collagen fibers in ovarian extracellular matrix

To evaluate collagen fibers in the extracellular matrix, sections were stained with picrosirius red (Abcam kit) as previously described (McKenzie *et al*., 2018). For each treatment, the percentage of area occupied by collagen fibers in ten different fields was measured using a camera attached to a microscope (Nikon, Eclipse, TS 100, Japan). Only collagen fibers were stained red with Picrosirius stain, while follicles remained unstained. Images were analyzed using Image J software (version 1.51p, 2017), and the circumference of unstained follicles was automatically excluded from the total area. The staining intensity of collagen fibers in the tissues was determined by measuring the average pixel intensity of the total area after background subtraction (Image J Software).

### Ovarian mouse mRNA expression of *SOD, CAT* and *NRF2*

Based on the results of follicle morphology, ovaries from mice treated with saline solution (1), 10.0mg/kg DOX alone, both DOX and 0.1mg/kg PIP, or 0.1mg/kg PIP were selected for the study of mRNA expression. According to the manufacturer’s instructions, total RNA was extracted using the TRIzol® method. Ovaries were first homogenized by the physical method using a scalpel blade under sterile conditions, 800 µL of Trizol® solution was added to each frozen sample and the lysate was aspirated through a 20-gauge needle before centrifugation at 10.000g for 3 minutes at room temperature. All of the lysates were then diluted 1:1 with 70% ethanol and applied to a mini-column that was provided in the kit. DNA digestion was performed with RNAse-free DNAse (340 K units/ml) for 15 min at room temperature after RNA was bound to the column. The RNA was eluted with 30 mL RNAse-free water after washing the column three times. RNA concentration was estimated by reading the absorbance at 260nm. Purity was checked at 280nm in a spectrophotometer (Amersham, Biosciences Cambridge, England). RNA samples were incubated at 70°C for 5 minutes and then cooled on ice before the reverse transcription reaction. Reverse transcription was performed in a total volume of 20µL consisting of 10µL sample containing 50ng RNA, 4µL reverse transcriptase buffer (Invitrogen), 8 units RNAsin, 150 units Superscript III reverse transcriptase, 0.036 U random primers, 10mM dithiothreitol, and 0.5mM each dNTP (Invitrogen). The mixture was incubated at 42.1°C for 1 hour, then at 80°C for 5 minutes, and finally stored at -20°C. The negative control was prepared using the same conditions but without adding reverse transcriptase.

For mRNA quantification, each real-time reaction (20µL) contained 10µL SYBR Green master mix (Applied Biosystems, Warrington, UK), 7.3µL ultrapure water, 1µL complementary DNA (cDNA) and 5 mM each primer. SOD, CAT, NRF2 and glyceraldehyde-3-phosphate dehydrogenase (GAPDH) were amplified (Table 1). Following previous studies (de Assis *et al*., 2023), GAPDH was used as a housekeeping gene to normalize mRNA expression. The melting curve of the PCR products was analyzed to confirm the specificity of each primer pair. A previously described protocol (Saetang *et al*., 2022) was used to verify the amplification efficiency for all genes. The thermal cycling profile for the first round of PCR was initial denaturation and polymerase activation for 10 minutes at 95°C, followed by 40 cycles of 95°C for 15s, 58°C for 30s, and 72°C for 30s, with a final extension of 72°C for 10 minutes. All reactions were carried out in a Step One Plus device (Applied Biosystems, Foster City, CA, USA). Negative control was prepared under the same conditions but without cDNA addition. Ct values were converted to mRNA expression levels using the 2^∆∆Ct method (de Assis *et al*., 2023).

**Table 1:** Primer pairs used for real-time PCR.

Target gene	Primer sequence (5’ → 3′)	Forward (F) Reverse (R)	GenBank accession no.
**GAPDH**	GAACGGATTTGGCCGTATTG GTGAGTGGAGTCATACTGGAAC	F R	GU214026.1
**SOD**	CTCGTCTTGCTCTCTCTGGTC CTTGCCTTCTGCTCGAAGTG	F R	NM_011434.2
**CAT**	CCAATGGCAATTACCCGTCC CCTTGTGAGGCCAAACCTTG	F R	NM_009804.2
**NRF2**	TTGCCCTAGCCTTTTCTCCG CTAGGAGATAGCCTGCTCGC	F R	NM_010902.4 R

### Statistical analysis

GraphPad Prism software was used for statistical analysis. Chi-squared test was used to evaluate the percentages of normal, primordial and developing follicles. Collagen fiber, stromal cell density, and SOD, CAT, and NRF2 mRNA expression data were evaluated by Kolmogorov-Smirnov test followed by Kruskal-Wallis test and Dunn multiple comparison test. Results are expressed as mean±standard error. When *p*<0.05, differences were considered significant.

## RESULTS

### Analysis of activation, survival and growth of ovarian follicles

**Figure 2:**
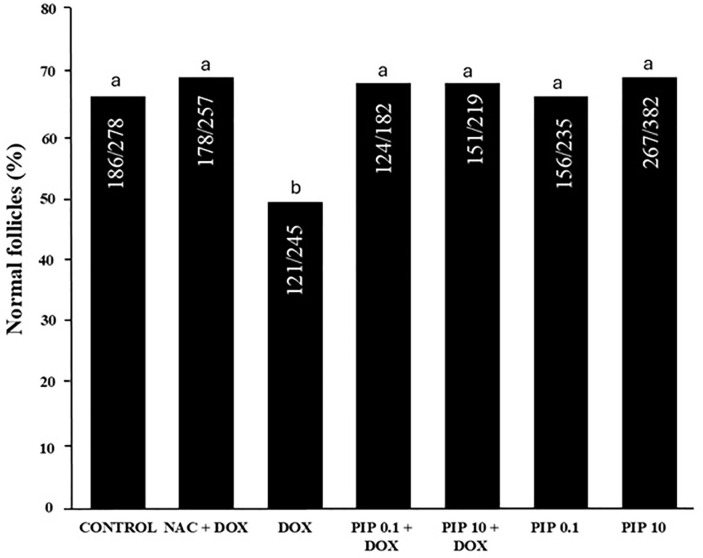
Percentage of normal and degenerated follicles evaluated by classical histology and hematoxylin and eosin staining. Chi-squared test was used to evaluate the percentages of normal follicles (*p*<0.05). a and b indicate statistically significant differences between treatments.

**Figure 3:**
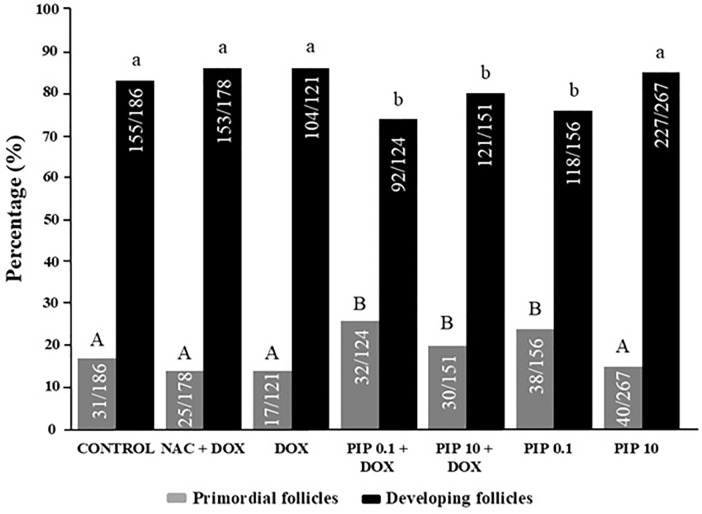
Percentage of primordial follicles and developing follicles. Chi-squared test was used to evaluate the percentages of primordial and developing follicles (*p*<0.05). aA and bB indicate statistically significant differences between treatments.

Mice treated with PIP alone (0.1 or 10.0mg/kg) had a similar percentage of morphologically normal follicles compared to the control group (Figure 2). On the other hand, mice treated with DOX alone had a significantly reduced percentage of normal follicles compared to the control group, but the administration DOX combined with PIP (0.1 or 10.0mg/kg) or NAC blocked the adverse effects of DOX and maintained the rate of normal follicles similar to untreated animals (Figure 2). Regarding follicular growth, mice treated with both DOX and PIP (0.1 or 10.0mg/kg) or with 0. mg/kg PIP alone had a higher percentage of primordial follicles and a lower percentage of developing follicles than the control group (*p*<0.05) (Figure 3).

### Assessment of collagen fibers stromal cells in the ovaries

**Figure 4:**
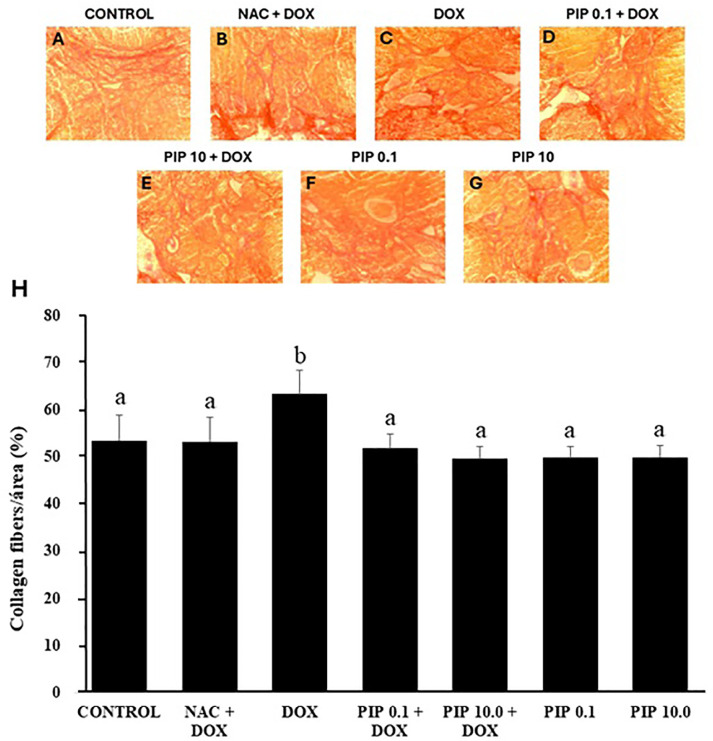
Morphology (A-G) and percentages (H) of collagen fibers area (mean±SD) in mice ovaries treated with NAC and DOX, DOX (10.0mg/kg), PIP (0.1 or 10.0mg/kg) or both PIP and DOX. Collagen fibers distribution was analyzed by the Kruskal-Wallis test, followed by Dunn’s comparison. a and b indicate statistically significant differences between treatments.

**Figure 5:**
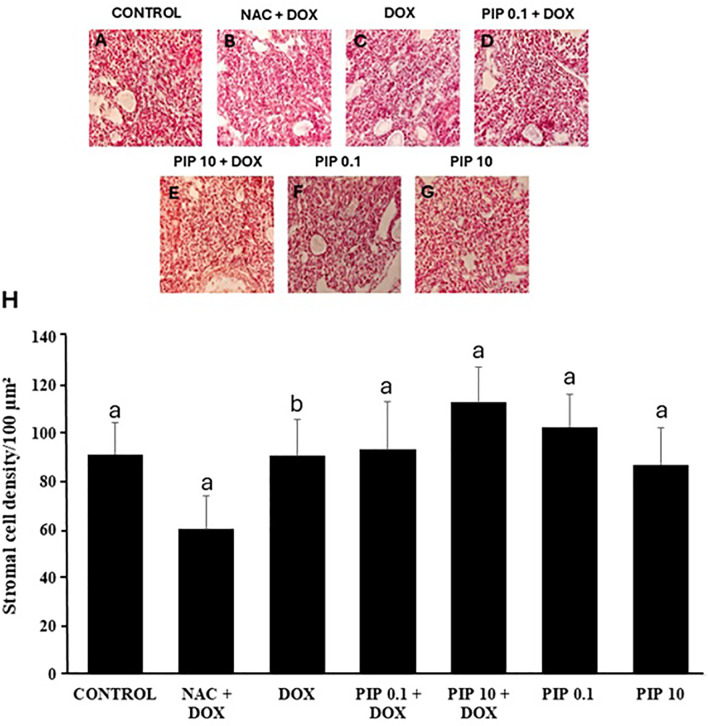
Morphology (A-G) and number (mean±S.E.M) of stromal cells (H) in mice ovaries treated with NAC and DOX, DOX (10.0mg/kg), PIP (0.1 or 10.0mg/kg) or both PIP and DOX. Stromal density was analyzed using the Kruskal-Wallis test, followed by Dunn’s comparison. a and b, different lowercase letters indicate statistically significant differences between treatments (*p*<0.05).

The DOX increased the area of collagen and fibrosis in mouse ovaries, but the combined treatment of DOX and PIP kept the collagen levels similar to untreated animals. Mice treated only with PIP or NAC also had the distribution of collagen fibers similar to untreated controls (Figure 4). Regarding to stromal cells, mice treated with both DOX and NAC had a reduced number of these cells, while those treated with a combination of DOX and PIP had similar number of stromal cells compared to control (Figure 5). Additionally, only NAC, PIP or DOX did not affect the number of stromal cells in the ovaries.

### Quantification of CAT, SOD and NRF2 mRNA expression in the ovaries

**Figure 6:**
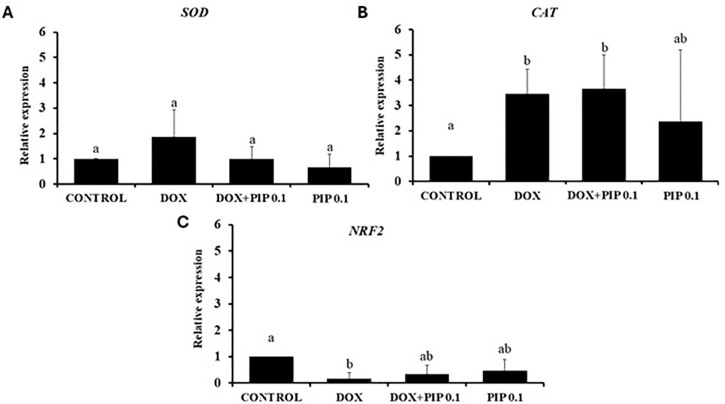
The levels of mRNA for (A) SOD, (B) CAT and (C) NRF2 in ovaries from control mice or treated with DOX (10.0mg/kg), both DOX and 0.1mg/kg PIP or only 0.1mg/kg PIP. Levels of mRNA for CAT, SOD and NRF2 were analyzed by the Kruskal-Wallis test, followed by the Dunn comparison. a, b different lowercase letters indicate significant difference between treatments (*p*<0.05).

The results showed that DOX, 0.1mg/kg PIP, or both DOX and 0.1mg/kg PIP did not affect the levels of SOD in mouse ovaries (Figure 6A). However, the levels of CAT mRNA were significantly higher in mice treated with both DOX and 0.1mg/kg PIP compared with those in the control group, but were not different from those in mice treated with DOX or 0.1mg/kg PIP alone (Figure 6B). DOX reduced the expression of NRF2 compared to control mice. However, there was no difference in the levels of mRNA for NRF2 in mice treated with DOX, 0.1mg/kg PIP, or both DOX and 0.1mg/kg PIP (Figure 6C).

## DISCUSSION

This study shows that PIP (0.1 or 10.0mg/kg) attenuates DOX-induced damage in mouse ovarian follicles. Previous studies have shown that PIP protects various cell types by attenuating oxidative stress (Saetang *et al*., 2022). The antioxidant activities of PIP occur through inhibition and/or reduction of ROS levels in chicken liver cells (Vurmaz & Atay, 2021). In rats, PIP reduced high-fat diet-induced oxidative stress by regulating the levels of SOD, CAT, glutathione peroxidase (GPx), glutathione-S-transferase (GST) and reduced glutathione (GSH) in different tissues, i.e. liver, heart, kidney, intestine and aorta (Echeverría *et al*., 2018). Furthermore, PIP exerts a chemopreventive effect in experimental lung carcinogenesis by modulating lipid peroxidation and increasing the activities of SOD, CAT, GSH and GPx (Selvendiran & Sakthisekaran, 2004).

In terms of follicular growth, DOX and PIP interacted to reduce the percentage of developing follicles. Follicular activation signals provided by the phosphatidylinositol 3-kinase (PI3K)/phosphatase and tensin homolog (PTEN)/Akt and mammalian target of rapamycin (mTOR) pathways stimulate follicular growth (Vo & Kawamura, 2021). Recently, both DOX and PIP have been shown to interact and suppress the PI3K/AKT/mTOR pathway, which may explain the inhibition of follicular growth (Hakeem *et al*., 2024).

In the present study, DOX reduced stromal cell density in mouse ovaries, but the presence of PIP attenuated these adverse effects. In this context, it is important to emphasize that chemotherapeutic drugs can have deleterious effects on the ovarian stroma (Spears *et al*., 2019). Ovarian stromal cells, which maintain tissue integrity and perform several critical functions, have a major impact on follicular development. In the ovary, DOX-induced toxicity results from the induction of oxidative stress and increased inflammatory response (Immediata *et al*., 2022). Therefore, we emphasize that PIP have antioxidant activity, a crucial factor in mitigating the adverse effects of DOX (Saetang *et al*., 2022) in ovarian stromal cells. Furthermore, no deleterious effects were observed when PIP was administered alone, suggesting a potential to prevent damage without affecting other structures.

The mice treated with DOX had a higher collagen area in their ovaries than those treated with PIP alone or in combination with DOX. The deleterious effects of DOX may be related to the release of inflammatory cytokines (Alves *et al*., 2022). In the pathogenesis of inflammatory diseases with tissue destruction, matrix metalloproteinases (MMPs) play a crucial role (Nissinen & Kähäri, 2014). A characteristic marker of cell death and scarring in ovarian tissue after chemotherapy was the presence of collagen fibrils in the cortical stroma (de Assis *et al*., 2023). Previously, PIP was shown to negatively regulate the expression of pro-inflammatory cytokines such as IL-1β and TNF-α (Azam *et al*., 2022). In this regard, excessive deposition of extracellular matrix components and ovarian fibrosis has been associated with reduced follicular density and ovarian dysfunction in women with ovarian endometriosis cysts, indicating a possible link between follicular loss and fibrosis (Lliberos *et al*., 2021).

In the present study, DOX reduced the mRNA levels for NRF2 and increased those of CAT in mouse ovaries, but PIP did not affect it. DOX also decreased NRF2 mRNA expression (de Assis *et al*., 2023). DOX produces high levels of ROS by inhibiting the expression and function of NRF2, a cellular redox homeostasis protein and master regulator of the antioxidant response. This may lead to increased oxidative damage and decreased antioxidant capacity, promoting oxidative stress, which has a positive correlation with lipid peroxidation and a negative correlation with increased antioxidant enzymatic activities (Sunitha *et al*., 2018). As a self-defense mechanism within the cell, the moderately increased expression levels of CAT after DOX administration suggest a self-defense response mechanism in the ovary (Niringiyumukiza *et al*., 2019). The levels of SOD and CAT can be reversed by treatment with spices such as pepper, ginger, and garlic, as they interfere with lipid peroxidation in biological systems, indicating the ability to eliminate free radicals (Vijayakumar *et al*., 2004). Therefore, in this study, the change in the expression of genes involved in antioxidant defense may have occurred due to the increase in ROS production after DOX treatment.

## CONCLUSIONS

The combined treatment of DOX and PIP (0.1 or 10.0mg/kg) preserved the integrity of follicles and collagen fibers in mouse ovaries. This data emphasizes the importance of integrated strategies to preserve the large pool of primordial follicles and allow the preservation of fertility in patients undergoing chemotherapy. The translation of these findings into clinical practice requires further research to confirm the safety and efficacy of PIP without compromising the therapeutic effects of chemotherapy.
